# Resistance induction based on the understanding of molecular interactions between plant viruses and host plants

**DOI:** 10.1186/s12985-021-01647-4

**Published:** 2021-08-28

**Authors:** Md. Shamim Akhter, Kenji S. Nakahara, Chikara Masuta

**Affiliations:** 1 Research Faculty of Agriculture, Hokkaido University, Sapporo, Hokkaido 060-8589 Japan; 2 Plant Pathology Division, Bangladesh Agricultural Research Institute (BARI), Joydebpur, Gazipur, 1701 Bangladesh

**Keywords:** Plant–virus interactions, Genome editing, RNA silencing, Plant activators

## Abstract

**Background:**

Viral diseases cause significant damage to crop yield and quality. While fungi- and bacteria-induced diseases can be controlled by pesticides, no effective approaches are available to control viruses with chemicals as they use the cellular functions of their host for their infection cycle. The conventional method of viral disease control is to use the inherent resistance of plants through breeding. However, the genetic sources of viral resistance are often limited. Recently, genome editing technology enabled the publication of multiple attempts to artificially induce new resistance types by manipulating host factors necessary for viral infection.

**Main body:**

In this review, we first outline the two major (*R* gene-mediated and RNA silencing) viral resistance mechanisms in plants. We also explain the phenomenon of mutations of host factors to function as recessive resistance genes, taking the *eIF4E* genes as examples. We then focus on a new type of virus resistance that has been repeatedly reported recently due to the widespread use of genome editing technology in plants, facilitating the specific knockdown of host factors. Here, we show that (1) an in-frame mutation of host factors necessary to confer viral resistance, sometimes resulting in resistance to different viruses and that (2) certain host factors exhibit antiviral resistance and viral-supporting (proviral) properties.

**Conclusion:**

A detailed understanding of the host factor functions would enable the development of strategies for the induction of a new type of viral resistance, taking into account the provision of a broad resistance spectrum and the suppression of the appearance of resistance-breaking strains.

## Background

Plants are sessile organisms that are continuously affected by numerous abiotic and biotic factors, directly impeding their growth or causing metabolic dysfunction [1–3]. For example, plants are often infected by pathogens such as fungi, bacteria, nematodes, and viruses. Viruses invade all forms of life, and viral infections cause physiological changes in the infected plants, leading to symptoms that result in significant yield loss. Viruses are undoubtedly difficult to control as they use the host cell machineries for infection. Viral diseases are major limiting factors for sustainable crop production worldwide. Although it is complicated to estimate the overall viral disease-related crop loss, it is estimated to be more than US$30 billion annually [4]. Recently, Akhter et al. [5] summarized the significant plant viral disease-related economic loss in important crops in Bangladesh. As obligate intracellular pathogens, viruses are exclusively dependent on the host cell machinery for their survival (e.g., multiplication and cell-to-cell movement), hence they alter host gene expression to suit their needs. Over the past decade, remarkable progress has been made in understanding the arms race between plants and viruses at the molecular level that could potentially provide new strategies useful for crop improvement programs. Plants with pinpointed disruption of host factors necessary for viral infection could become highly resistant. In this case, the effect of genetic modification on plant growth could be minimized. Furthermore, it might be possible to design plants that do not allow the emergence of resistance-breaking strains. In the following sections, we will outline the known mechanisms of virus resistance in plants, then discuss the newly discovered underlying molecular interactions between the host and viral factors and introduce the possibility of virus resistance induction through modifying plant–virus interaction(s).

## Main text

### Plant–virus interaction-related molecular mechanisms

#### Historical perspective of molecular plant–virus interactions

Recently developed techniques in plant virology on RNA silencing, such as virus-induced gene silencing, large-scale genomic analysis, and epigenetic analysis, have enriched the understanding of viral pathogenicity and host responses in antiviral resistance. Plant antiviral activities include *R* gene-mediated resistance, recessive resistance, and antiviral RNA silencing [6, 7]. *R* gene-mediated resistance, the most intensively studied resistance mechanism against bacteria and fungi, generally accompanying hypersensitive response (HR), is also effective in viruses.

The *N* gene (*N*), isolated from *Nicotiana glutinosa*, is the first identified virus-related *R* gene. The avirulence (Avr) protein recognized by the N protein against tobacco mosaic virus (TMV) is the viral 126-kDa protein (126 k). The molecular interaction between N and 126 kDa induces HR-based resistance and subsequently systemic acquired resistance (SAR), supporting the gene-for-gene theory [8, 9]. When *N*-carrier tobacco plants are infected with TMV, they accumulate salicylic acid (SA), which then induces the expression of the defense-related genes and contributes to the development of SAR in the non-infected parts of the infected plants [10]. Multiple examples for the *R* genes and their corresponding Avr factors and virus-interacting proteins have been reported for different plant species (Table 1). To explain the sequential interactions between hosts and pathogens, Jones and Dangl [11] proposed the zig-zag model in 2006. In their model, the plant immune system comprises two defense response layers: pathogen-associated molecular pattern (PAMP)-triggered immunity (PTI) and effector-triggered immunity (ETI). PTI represents a basic defense mechanism by preventing pathogen invasion in response to specific structures or proteins associated with the pathogen, defined as the so-called PAMPs or microbe-associated molecular patterns. Plants show susceptibility only when a pathogen successfully achieves both the suppression of the PTI response and the production of its pathogenic effectors. ETI, the second level of the defense response, is triggered when the R gene products directly or indirectly detect the presence of specific effectors. Consequently, an effective ETI would keep plants resistant but insufficient ETI could lead to disease establishment (susceptibility). To explain host–pathogen interactions in *R* gene-mediated resistance, the guard hypothesis and the decoy model have been proposed in multiple pathosystems [11–13]. Due to the intracellular parasitic viral nature, which absolutely requires a live host cell machinery, any common fungal and bacterial resistance model would not fit viral resistance. Pattern recognition receptors (PRR), which serve as a major defense element by triggering the first layer of resistance [14], cannot play a role in fighting against plant viruses as viruses do not express extracellular PAMPs. However, in the modified zig-zag model [15], RNA silencing is regarded as a major antiviral mechanism for PTI and viral RNA silencing suppressors (RSSs) are regarded as effectors to overcome host RNA silencing. RSSs are then recognized by ETI as a virulence proteins [15].

**Table 1:** Dominant plant virus resistance genes in different host plant species and Avr/viral proteins inducing resistance

Virus^a^	Avr or viral protein inducing resistance^b^	Host Plant	Resistant gene	Resistant protein	Type of resistance^c^	References
TCV	CP	*Arabidopsis thaliana*	*HRT*	CC-NB-LRR	HR	[16]
CMV	CP	*A. thaliana*	*RCY1*	CC-NB-LRR	HR	[17]
PIAMV	(–)	*A. thaliana*	*JAX1*	Jacalin-like lectin	Blocking RNA accumulation	[18]
PVX	RdRP	*A. thaliana*	*JAX1*	Jacalin Family	Blocking systemic movement	[19]
TEV	(–)	*A. thaliana*	*RTM3*	MATH-containing protein	Systemic resistance	[20]
CaMV	P1	*A. thaliana*	*CAR1*	Not identified	HR	[21]
TuMV	UN	*Brassica compestris*	*BcTUR3*	TIR-NB-LRR	Systemic resistance	[22]
TuMV	UN	*B. compestris*	*TuRB07*	CC-NB-LRR	Extreme resistance	[23]
TMV	CP	*Capsicum annuum*	*L locus*	CC-NB-LRR	HR	[24]
PRSV	(–)	*Cucumis melo*	*Prv (Muti alleles)*	TIR-NB-LRR	HR	[25]
PepMMoV, PepSMV, PVY	RdRp	*C. annuum*	*Prv4*	CC-NB-LRR	HR	[26]
CLRDV	Po	*Gossypium hirsutum*	*cbd*	TIR-NB-LRR	HR	[27]
CABMV, TuYV, PLRV	Po	*Nicotiana glutinosa*	*RPO1*	NB-LRR	HR	[28]
ToYLCV	V1 and C3	*Solanum chilense*	*Ty1/Ty3 (Multi allelle)*	RDR	RNA silencing	[29]
ToYLCV	Rep/C1	*S. habrochites*	*Ty2*	CC-NB-LRR	HR	[30]
TSWV	NSm	*S. lycopersicum*	*5w5b*	NB-ARC-LRR	HR	[31]
TSWV	NSm	*N. alata*	*RTSW*	CC-NB-LRR	HR	[32]
PVX	CP	*S. tuberosum*	*Rx1*	CC-NB-LRR	Translation arrest	[33]
PVY	CP	*S. stoloniferum*	*Rysto*	TIR-NB-LRR	HR	[34]
MYMV, BCMV	(–)	*Vigna mungo*	*CYR1*	CC-NB-LRR	HR	[35]

^a^TCV, turnip crinkle virus; CMV, cucumber mosaic virus; PIAMV, plantago asiatica mosaic virus; PVX, potato virus X; TEV, tobacco etch virus; CaMV, cauliflower mosaic virus; TuMV, turnip mosaic virus; TMV, tobacco mosaic virus; PRSV, papaya ringspot virus; PepMMoV, pepper mild mottle virus; PepSMV, pepper severe mosaic virus; PVY, potato virus Y; CLRDV, cotton leaf roll dwarf virus; CABMV, cucurbit aphid borne mosaic virus; PLRV, potato leaf roll virus; ToYLCV, tomato yellow leaf curl virus; TSWV, tomato spotted wilt virus; MYMV, mungbean yellow mosaic virus; BCMV, bean common mosaic virus

^b^Proteins that can bind to R proteins either directly or indirectly. CP, coat protein; RdRP, RNA-dependent RNA polymerase; Po, suppressor of RNA silencing; V1, coat protein of ToYLCV; C1, replication associated protein; C3, replication enhancer protein; Nsm, Non-structural movement protein; (–), Not reported

^c^HR, hypersensitive response

Recessive resistance is often due to modifications in a certain gene, encoding a host factor critical for viral infection [36]. Recessive resistance might sometimes be provided by a deficiency in a negative regulator for plant defense. For example, several deficient genes of the eukaryotic translation initiation factor (eIF) 4E, eIF4G, and their isoforms are the most widely exploited recessive resistance genes in various plant species and are indeed effective against a subset of viral species [37]. High throughput sequence and genome editing technologies greatly contributed to enhancing plant genetic resources for breeding in various crop species. Multiple recessive resistance genes have been identified in various plant–virus interactions (Table 2).

**Table 2:** Recessive reistance genes against plant viruses and viral proteins involved in resistance in different host plant species

Virus^a^	Viral protein involved in resisance^b^	Host plant	Gene/ Locus	Remarks	References
BCMV	VPg	*Phaseolus vulgaris*	*bc3*	eIF 4E (mutagenesis)	[38]
CIYVV	Vpg	*Pisium sativum*	*cyv1/cyv2*	eIF4E ( mutagenesis)	[39]
TEV &PVY	VPg	*Capsicum spp.*	*pvr1/pvr2*	eIF4E ( mutagenesis)	[40]
RYMoV	Unknown	*Oryza sativa*	*rymv2*	*CPR5* homolog	[41]
BCMV	Unknown	*P. vulgaris*	*bc3*	eIF4E	[42]
BaMMV	VPg	*Hordeum vulgare*	*rym7*	eIF(iso)4E	[43]
TuMV	Unknown	*Brasssica juncea*	*retr03*	Mutation of eIF2Bβ	[44]
PIAMV	TGB2, TGB3	*Arabidopsis thaliana*	*?*	Mutation of nCBP	[45]
CBSV and UCBSV	VPg	*Manihot esculenta*	*?*	Mutation of 4E (eIF4E) and nCBP	[46]
PVY	VPg	*Nicotina tabacum*	*va*	eIF4E (iso)	[47]
YoMV, ToMV, TMV, TMGMV, PMMoV		*Nicotiana spp., Solanum lycopersicum, C. annuum, O. sativa*	*TOM1; TOM3*	EMS mutagenesis	[37, 48]
PepLCIV, PepLCAV		*C. annuum*	*pepy1*	Silencing of *CaPelota*	[49]
ToMV, YoMV		*A. thaliana, N. tabacum*	*ARL8*	Simultaneous mutation of *ARL8a* and *ARL8b* by T-DNA insertion	[37, 50]
TuMV, PPV		*A. thaliana, N. tabacum, Zea mays, O. sativa, Mesembryanthemum crystallinum*	*DBP1*	T-DNA mutant	[51]
WMV, PPV, BaMV		*S. lycopersicum, S. tuerosum, Populus trichocarpa, Sorghum bicolor, O. sativa, Tritichum aestivum, Z. mays*	*cPGK*	Natural resistance gene, rwm1 in *Arabibopsis thaliana* CVI-0 ecotype	[37, 52, 53]
PIAMV, PVX, AltMV		*A. thaliana, O. sativa, S. lycopersicum*	*EXA1*	EMS mutagenesis	[54]
TuMV		*A. thaliana, P. sativum, N. benthamiana*	*PVIP1, PVIP2*	Knockdown mutant of each PVIP	[55]
GFPV, CaMV		*A. thaliana*	*PDLP1, PDLP2,PDLP3*	Triple mutant of *PDLP1*, *PDLP2* and *PDLP3* by T-DNA insertion	[56]
TuMV		*A. thaliana*	*PCaP1*	T-DNA mutant	[57]
CaLCuV, TVCV, TuMV		*A. thaliana*	*SYTA*	T-DNA mutant	[58, 59]
TuMV		*A. thaliana*	*Sec24a*	EMS-induced mutant	[60]
TSWV		*A. thaliana*	*RHD3*	T-DNA mutant	[61]
BaYMV, BaMMV		All plant species	*PDIL5-1*	Natural resistance gene, rwm11 in barley	[62]
TuMV		All plant species	*IRE1*	Double mutation of *IRE1a* and *IRE1b* by T-DNA insertion	[63]
TuMV, PVX		All plant species	*bZIP60*	T-DNA mutant	[37, 64]
CMV		*A. thaliana*	*HAT1, HAT2, HAT3*	Triple mutant by the HAT genes	[65]

^a^ Abbriviations are explained in Table 1

^b^Viral proteins, which are involved in the resistance associated with eIF4E family proteins, are shown

As viruses are intracellular parasites containing either RNA or DNA genomes in a virion, RNA silencing is considered a major antiviral mechanism [15, 66]. Successful antiviral RNA silencing results in the degradation of the viral genome at the initial infection site [67]. In addition, several other viral resistance mechanisms have also been reported. These include mechanisms related to the ubiquitin–proteasome machinery, autophagy, and DNA methylation [29, 68, 69]. One interesting example is a tobacco calmodulin-like protein, rgs-CaM, leading to the autophagy-mediated degradation of viral RSSs [70–72].

### Resistance conferred by the interactions between R genes and plant viruses

Host *R* genes typically induce race-specific resistance in response to the *Avr* genes of pathogens [73, 74]. When plant–virus interactions occur in a single cell, an *R* gene triggers an HR response, a form of programmed cell death that rapidly kills infected cells and restricts the viral invasion. HR is generally associated with various molecular events: the activation and expression of salicylic (SA), jasmonic acid (JA), mitogen-activated protein kinase signaling, calcium ion influx, callose deposition at the plasmodesmata, membrane permeability modification, pathogenesis-related (PR) protein expression, and immediate accumulation of reactive oxygen species (ROS) and nitric oxide (NO) [75, 76]. The majority of the *R* genes encode nucleotide-binding (NB) and leucine-rich-repeat (LRR) domains but the Avr proteins do not share any common structure [11]. For the NB-LRR proteins, three domains consist of the center nucleotide-binding site (NBS), an LRR at the C terminal end, and a coiled-coil or Toll and human interleukin receptor (TIR) domain at the N-terminus [77]. The NBS domain also contains the Apaf-1/R protein/CED4 (ARC) domain, thought to be a molecular switch regulating R protein activation through ATP hydrolysis-related signal transduction [78–81]. The N-terminus displays an important role in the specific interaction with an Avr factor [78]. The NB-LRR-mediated Avr effector recognition, initiating the downstream defense responses, could occur both directly, and indirectly mediated by cellular cofactors.

### Resistance conferred by antiviral RNA silencing

RNA silencing, also known as RNA interference (RNAi) or post-transcriptional gene silencing, is a host plant counter-defense against virus-derived double-stranded (ds) RNA [82–84]. In the plant–virus interactions, the most common host defense against viruses is considered to be RNA silencing. Antiviral RNA silencing is triggered by viral dsRNA generated either by replication intermediates or by secondary intramolecular RNA folding (hairpin) in the host cells [85, 86]. In these cells, the viral dsRNAs are cleaved by Dicer-like (DCL) enzymes into virus-induced small RNAs (vsRNAs) [87]. vsRNAs are then incorporated into the RNA-induced silencing complex and guide Argonaute (AGO) proteins to the targeted RNA for degradation or translational arrest [88]. vsRNA, as the antiviral RNA silencing signal, is subjected to the secondary amplification of sRNAs by RNA-dependent RNA polymerase (RDR) 6 and transferred through the plasmodesmata and phloem, inducing systemic viral defense [67]. DNA viruses are also subjected to antiviral RNA silencing [89]. In the co-evolutionary plant–virus interaction context, viruses might have acquired counter-defense mechanisms by suppressing host antiviral silencing [87]. To date, a considerable number of viral RSSs have been already reported [90]. Increasing evidence shows that plants have actually evolved certain mechanisms to fight viral RSSs, regarded as counter-counter-defense responses in the molecular arms race [88]. Based on the understanding of the RNA silencing mechanisms, we can practically make exogenous applications of viral dsRNA and siRNA for disease protection [91]. Even virus resistance in a transgenic plant expressing a viral sequence is operated by RNA silencing. For example, the coat protein (CP)-mediated transgenic papaya resistance against papaya ringspot virus through RNA silencing is one of the success stories in commercial application [92]. Exogenous tomato spotted wilt virus (TSWV)-derived dsRNA application for virus resistance induction in tobacco indicates a promising prospect of spray-induced gene silencing for plant–virus interactions [93].

### Resistance conferred by natural variants and manipulation of host susceptible factors for virus infection

Due to their intracellular parasitic nature, viruses are dependent on the host cellular mechanisms for their survival. After the viral entry into the plant cells, the viral genome is released from the capsid, then viral proteins are translated. Due to a limited number of viral-encoded genes, viruses require numerous host factors to pursue a successful infection cycle consisting of replication, transcription, and translation, as well as cell-to-cell and long-distance movement [94]. The absence or modification of a host factor necessary for the virus infection cycle is regarded as an efficient defense approach and is considered a form of passive resistance. Such passive resistance generally exhibits recessive inheritance. For example, eIF4E is a key player in the translation initiation by recruiting messenger RNAs to the ribosomal complex and has been repeatedly identified as an essential host factor for viral infection [94]. Natural variation of eIF4E can confer resistance to crops against potyviruses; the modification of host factors could thus be a common target to develop resistant varieties [95–97]. The known recessive resistance genes are summarized in Table 2.

### Manipulation of host factors confer viral resistance

#### How to manipulate host factors: lessons from the manipulation of eIF4E family genes

Until the development of genome editing techniques, site-directed mutagenesis had not been available in plants. T-DNA insertion lines and chemical mutagen-based, such as ethyl methanesulfonate (EMS), random mutagenesis had been an alternative. We present an example of how *eIF4E* family gene mutations lead to virus resistance. Among five eIF4E family members (eIF4E, eIF4E1b, eIF4E1c, eIF (iso) 4E, and the novel cap-binding protein [nCBP]) in *Arabidopsis thaliana*, eIF4E, and eIF (iso) 4E are reportedly involved in potyvirus infection [98]. In the inoculation tests using the homozygotes of the null alleles, clover yellow vein virus (ClYVV) was found to use eIF4E while turnip mosaic virus (TuMV) uses eIF(iso)4E [99]. Resistance-breaking isolates of TuMV could infect the plant with a single null allele of *eIF4E* or *eIF (iso) 4E* as these isolates could use both eIF4E and eIF (iso) 4E [100]. Although *eIF4E* and *eIF(iso)4E* double mutants are not produced due to their fatality, Bastet et al. [100] produced an alternative resistant plant to the resistance-breaking TuMV strains by pyramiding the null allele of *eIF(iso)4E* and the base-edited allele of *eIF4E*, mimicking the *eIF4E* resistance allele in pea.

Until recently, nCBP, another eIF4E isoform that is genetically distant to eIF4E and eIF(iso)4E, was not a susceptible factor for viral infection. However, viruses distinct from potyviruses reportedly use nCBP [45, 46]. T-DNA insertion lines for the *nCBP* of *A. thaliana* were impaired in the cell-to-cell movement of plantago asiatica mosaic virus, a member of the genus *Potexvirus*, by inhibiting the expression of the viral movement protein [100]. In cassava plants, the *eIF4E* family consists of five members (*eIF4E*, two *eIF(iso)4Es*, and two *nCBPs*). Cassava brown streak virus and Ugandan cassava brown streak virus, members of the genus *Ipomovirus*, are the causal agents of the cassava brown streak disease. The viral genome-linked proteins (VPg) of these viruses have a higher affinity to nCBPs than eIF4E and eIF(iso)4E. Simultaneous CRISPR/Cas9-mediated genome editing of two *nCBPs* genes reduced the susceptibility to these viruses in cassava and the severity of symptoms caused by these viruses [46].

#### Natural variation and in-frame deletion of eIF4E1 outstrip the null allele for viral resistance in tomato

Creating a null allele of a susceptible factor to a virus represents the risk of a potentially detrimental effect on plant growth if the given factor is also essential for the plant. However, for functionally redundant factors, emerging resistance-breaking viruses represent another risk, potentially switching the factor in use from a null to a redundant allele. Moreover, in terms of conferring antiviral resistance, the functional alleles of *eIF4E1* carrying non-synonymous base substitutions or a small in-frame deletion reportedly outstripped the null allele in tomato plants [101, 102]. Tomato exhibits two *eIF4Es*, *eIF4E1* and *eIF4E2*, *eIF(iso)4E,* and *nCBP*. The natural *eIF4E1* allele, *pot1*, isolated from a wild tomato relative (*Solanum habrochaites*), reportedly exhibits a wider resistance spectrum against potato virus Y and tobacco etch virus strains than the corresponding null allele. The null allele was obtained by the TILLING approach with EMS-mediated randomly mutated tomato plants [103]. Further analysis demonstrated that the wider resistance spectrum by *pot1* was comparable to that by an *eIF4E1* and *eIF4E2* double mutant, suggesting that pot1 lacks the function to support viral infection but can compete with eIF4E2 or inhibit its interaction with viruses [102]. The growth defect observed in the double mutant demonstrates the additional usefulness of *pot1* in tomato production.

Recently, we edited *eIF4E1* by CRISPR/Cas9 and obtained three alleles, including a nucleotide insertion (*1INS*) and nine nucleotide deletion (*9DEL*) within the eIF4E1 protein coding region [101]. *1INS*, containing a frameshift, is considered to be a null allele, and its homozygote showed resistance to the N strain of potato virus (PVY^N^). *9DEL* would be a functional allele though it lacks three amino acids. The fact that no significant resistance to PVY^N^ was observed in the *9DEL* homozygotes indicates that 9DEL retains some function at least partially in PVY^N^ infection. Unexpectedly, the *9DEL* homozygote but not *1INS* showed partial resistance to cucumber mosaic virus (CMV), suggesting that the modified function of 9DEL could interfere with CMV infection. Considering the above observations, functional alleles with base-editing or in-frame indels could be occasionally very effective for crop production against viruses.

#### Modified plant–virus interactions (MPVI)-mediated antiviral resistance

Genome editing, silencing in transgenic plants, and random mutagenesis mostly result in (partial) loss of function of a particular gene. The primary target genes to confer antiviral resistance would be host susceptible factors, which contribute to viral infection, multiplication, and spread. We list a number of these susceptible factors in Table 2. Host susceptible factors were exhaustively identified using yeast as a host for a plant virus. Two plant viruses, brome mosaic virus and tomato bushy stunt virus, were studied by two research groups [104, 105]. Both studies identified more than a hundred genes affecting virus accumulation, but few were shared in the identified genes between the two studies, indicating that each virus distinctly uses host factors.

In conferring virus resistance by manipulating host factors necessary for the virus, we must understand the viral infection cycle in detail, because some viruses use unusual host factors. There may be inconsistencies in the newly found functions of host factors, given their original functions. For example, as described in the former section, AGO1 is reportedly a core component of RNA silencing as a slicer of its target RNA and involved in antiviral defense. However, AGO1 was recently reported to interact with HC-Pro of potato virus A [106]. This interaction facilitates systemic infection of potato virus A by stabilizing the viral coat protein to form viral particles [107]. Similarly, a receptor-like kinase, BAM1 was shown to be located at plasmodesmata to facilitate the systemic spread of RNA silencing in *A. thaliana* while it is also a target protein of C4, an RNA silencing suppressor of tomato yellow leaf curl virus [108]. In addition, BAM1 was shown to bind to the movement protein of TMV and promote the cell-to-cell movement of TMV at an early stage of infection in *N. benthamiana* [109]. rgs-CaM is reportedly an endogenous RNA silencing suppressor [110], but it works for the defense against CMV by binding to and directing degradation of the viral RNA silencing suppressor 2b under activation of SA signaling [70–72, 111].

Although RDR1 and DCL4 are reportedly involved in small RNA biogenesis in antiviral RNA silencing, the loss of function mutation of *RDR1* and silencing of *DCL4* reduced susceptibility to viruses and potato spindle tuber viroid in *N. benthamiana* [112, 113]. RDR1 and DCL4 are not visibly susceptible host factors necessary for viruses and viroids but their absence may enhance the RDR6- and DCL2,3-mediated anti-virus and anti-viroid defenses, respectively. In other words, competitive interactions among the redundant RDRs and DCLs may result in these inconsistent host reactions. Another inconsistent host reaction with a susceptible factor was reported recently; hiper-susceptibility to TuMV was observed in the *eIF4E* null mutant of *A. thaliana* though the *eIF4E* mutant is resistant to ClYVV [114]. Recently autophagy has been reported to be involved in both antiviral and proviral mechanisms [115–117], suggesting that plants use their gene products flexibly.

Even the defense-related genes can become an effective target to induce antiviral resistance; the genetic resources for antiviral resistance might exceed our expectations. To explore promising host factors and to find how to edit them for the modified-plant–virus interaction (MPVI)-mediated antiviral resistance, more knowledge, and research is necessary.

### Molecular mechanisms for antiviral resistance induced by plant activators

Induction of plant resistance, which is achieved either by chemicals (SAR) or by microbes (induced systemic resistance, ISR), is an alternative to manage viral diseases in crops [118]. Phytohormones such as SA, JAs, ethylene (Et), and abscisic acid (ABA) reportedly regulate plant responses against pathogens [119]. A comprehensive list of chemicals to plant viruses has been listed in Table 3. Auxins (Auxs), brassinosteroids (BRs), cytokinins (CKs), and ABA are known for their roles in plant growth and development but have been recently documented to also play a role in plants-virus interactions [120–122]. Interestingly, SA, JA, and Et, which regulate the defense pathways, exhibit antagonistic interactions with each other. For example, the activation of the SA signaling pathway can repress the JA/Et pathway mainly through the two genes, *NPR1* (*NONEXPRESSER OF PATHOGENESIS RELATED GENE 1*) and *WRKY70*, and the ABA pathway through *NPR1* or its downstream elements [123–126]. Conversely, the activation of the JA/Et pathway represses the expression of certain genes downstream of the SA signaling via *MAPK4* (*MITOGEN‐ACTIVATED PROTEIN KINASE 4*) and *JIN2* [124, 127]. However, the SA biosynthesis and its signaling are triggered after viral effectors are recognized by the R proteins that lead to the incompatible interaction. The activation of the incompatible interaction results in host responses to restrict virus spread from the infection site by inducing HR and the accumulation of ROS and PR proteins [128, 129]. SA is also responsible for the activation of SAR in distal tissues, which minimizes the damage of secondary attacks by the pathogen. In tomato plants, the exogenous application of SA triggers the expression of the *SlPR1* gene, inducing resistance to TYLCV [130].

**Table 3:** Plant activators and microbes that induce host resistance against plant viruses

Plant activator and microbe^a^	Virus^b^	Host	Resistance	Hormones^c^	Reference
ASM	PIAMV, PVX, TuMV, CCYV	*Nicotiana benthamiana,Cucumis melo*	Systemic	SA	[131, 132]
BABA	TMV	*N. tabacum*	HR	SA	[133]
Probenazol	TMV	*N. tabacum*	HR	SA	[134]
	TMV	*N. benthamiana*	Systemic	BR	[135]
Brassinosteroid	RSV	*Oyaza sativa*	Systemic	JA/BR	[136]
	CMV	*Cucurbita pepo*	Systemic	BR	[137]
Chitosan	TBSV	*Phaseolus vulgaris*	HR	SA	[138]
Quassinoids	TMV	*N. tabacum, N. glutinosa*	Systemic, HR	Not reported	[139]
Quinolizidine alkaloids	TMV	*N. tabacum*	Systemic	Not reported	[140]
Harpin popW	TMV	*N. tabacum*	HR	SA	[141]
Soluble silicon	TRSV, TMV	*N. tabacum*	Systemic	SA	[142]
Esterified whey protein fractions (EWPF)	TMV	*N. tabacum*	HR	SA	[143]
Eudesmanolides	TMV	*N. tabacum, N. glutinosa*	Systemic, HR	SA	[144]
Spermine and longer polyamines	CMV	*Arabidopsis thaliana*	Systemic	SA	[145]
PABA	TMV	*Capsicum annuum*	Systemic	SA	[146]
Eugenol	TYLCV	*Solanum lycopersicum*	Systemic	SA	[147]
Ningnanmicin	TMV	*N. tabacum*	Systemic	SA	[148]
SHAM	CMV	*A. thaliana*	Systemic	SA	[149]
Ascorobic acid	TuMV	*Brassica rapa*	Systemic	JA	[150]
2,3-butanediol	CMV, TMV	*C. annuum*	Systemic	SA/JA/ET	[151]
*Bacillus amyloliquefaciens* strain MBI600	TSWV, PVY	*S. lycopersicum*	Systemic	SA	[152]
*Bacillus amyloliquefaciens* strain 5B6	BBWV, CMV, PepMoV	*C. annuum*	Systemic	SA / JA	[153]
*Penicillium simplicissimum*	CMV	*A. thaliana, N. tabacum, N. benthamiana*	Systemic	SA/ JA/ET	[154]
*Trichoderma harzianum* strain T-22 (T22)	CMV	*S. lycopersicum*	Systemic	SA/JA/ET	[155]
*Pseudozyma churashimaensis* (Yeast)	CMV, PeMMoV, PeMoV, BBWV	*C. annuum*	Systemic	SA/JA/ET	[156]

^a^ASM, acibenzolar-S-methyl; BABA, β- aminobutyric acid; SHAM, salicylhydroxamic acid; PABA, para-aminobenzoic acid

^b^Abbreviations are explained in Table 1

^c^BR, brassinosteroid; SA, salisylic acid; JA, jasmonic acid; ET, ethylene

## Conclusion

Modifying the host factors necessary for the virus is interesting to potentially confer viral resistance in plants but no such simple solution is available in reality. For example, if the function of the target host factor is not well understood, the knockout of the corresponding gene might also negatively affect the growth and development of the plant. Until recently, it was impossible to specifically eliminate a target host factor. However, with the recent development of genome editing technologies, we are now able to specifically edit various host factors, and some of the resulting edited plants acquire unexpected viral resistance not only to the target virus but also to other viruses. Therefore, the possibility of producing new virus-resistant crops by specifically manipulating host factors based on a good understanding of their functions should be extensively explored.

## Data Availability

Not applicable.

## Abbreviations

eIF4E: Eukaryotic translation initiation factor 4E
HR: Hypersensitive response
Avr: Avirulence
SAR: Systemic acquired resistance
TMV: Tobacco mosaic virus
PAMP: Pathogen-associated molecular pattern
PTI: Pathogen triggered immunity
ETI: Effector-triggered immunity
PRR: Pattern recognition receptors
RSSs: Viral RNA silencing suppressors
rgs-CaM: Regulator of gene silencing calmodulin related protein
JA: Jasmonic acid
PR: Pathogenesis-related
ROS: Reactive oxygen species
NO: Nitric oxide
NB: Nucleotide-binding
LRR: Leucine-rich-repeat
NBS: Nucleotide-binding site
TIR: Toll and human interleukin receptor
Apaf-1: Apoptotic protease-activating factor-1
CED 4: *Caenorhabditis elegans* Death-4
ATP: Adenosine triphosphate
RNAi: RNA interference
dsRNA: Double-stranded RNA
DCL: Dicer-like enzyme
AGO: Argonaute
vsRNA: Viral small RNA
RDR: RNA-dependent RNA polymerase
siRNA: Short interfering RNA
CP: Coat protein
TSWV: Tomato spotted wilt virus
T-DNA: Transfer DNA
EMS: Ethyl methanesulfonate
nCBP: Novel cap-binding protein
CIYVV: Clover yellow vein virus
TuMV: Turnip mosaic virus
VPg: Viral protein genome linked
CRISPR/Cas9: Clustered regularly interspaced short palindromic repeats/CRISPR-associated protein 9
*pot1*: Protection of telomere 1
TILLING: Target induced local lesions in genomes
1INS: One nucleotide insertion
9 DEL: Nine nucleotides deletion
PVY: Potato virus Y
CMV: Cucumber mosaic virus
MPVI: Modified plant–virus interactions
HC-pro: Helper component protease
BAM1: Barely any meristem1
SAR: Systemic acquired resistance
ISR: Induced systemic resistance
Et: Ethylene
ABA: Abscisic acid
BR: Brassinosteroids
CKs: Cytokinins
*NPR1*: *Nonexpresser of pathogenesis related gene *1
*MAPK*: *Mitogen-activated protein kinase*
*SIPR1*: *Solanum lycopersicum pathogenesis related gene* 1
TYLCV: Tomato yellow leaf curl virus
TCV: Turnip crincle virus
PIAMV: Plantago asiatica mosaic virus
PVX: Potato virus X
TEV: Tobacco etch virus
CaMV: Cauliflower mosaic virus
PRSV: Papaya ringspot virus
PepMMoV: Pepper mild mottle virus
PepSMV: Pepper severe mosaic virus
CLRDV: Cotton leaf roll dwarf virus
CABMV: Cucurbit aphid borne mosaic virus
PLRV: Potato leaf roll virus
MYMV: Mungbean yellow mosaic virus
BCMV: Bean common mosaic virus
Nsm: Non-structural movement protein
ASM: Acibenzolar-S-methyl
BABA: β-Aminobutyric acid
SHAM: Salicylhydroxamic acid
PABA: Para-aminobenzoic acid
